# Subspecialisation in radiology in Europe, a survey of the accreditation council of imaging

**DOI:** 10.1186/s13244-023-01481-y

**Published:** 2023-09-25

**Authors:** Mitja Rupreht, Paolo Ricci, Helmut Prosch, Miraude E. A. P. M. Adriaensen

**Affiliations:** 1 Radiology Department, UMC Maribor, Ljubljanska 5, 2000 Maribor, Slovenia; 2 Medical Faculty, University of Maribor, Maribor, Slovenia; 3 Department of Radiology and Nuclear Medicine, Maastricht UMC+, Maastricht, The Netherlands; 4 Department of Radiological, Oncological and Pathological Sciences, Sapienza University of Rome, Rome, Italy; 5 Department of Biomedical Imaging and Image-Guided Therapy, Medical University of Vienna, Vienna, Austria; 6 Department of Medical Imaging, Zuyderland Medical Center, Sittard-Geleen, Heerlen, Brunssum, Kerkrade, The Netherlands

**Keywords:** Subspecialisation, Radiology, European subspecialty diploma, Recognition, Harmonisation

## Abstract

**Background:**

To provide an overview of existing Subspecialty Exams and Diplomas in Radiology and their endorsement as well as to providing an insight into the status of subspecialisation in radiology in Europe. The European Training Curriculum for Subspecialisation in Radiology mentions thirteen fields of subspecialisation within radiology. The websites of the corresponding subspecialty societies were checked for Subspecialty Exams and Diplomas. In addition, we performed a survey among European radiologists regarding subspecialisation in radiology.

**Results:**

Ten out of 13 European subspecialty societies offer a European subspecialty diploma. At least 7 out of the 10 European subspecialties societies in radiology offering a European subspecialty diploma obtained European Society of Radiology (ESR) endorsement. Two out of 10 obtained European Union of Medical Specialists—Council of European Specialist Medical Assessment endorsement. Survey among European radiologists who were ESR full members in March 2021 demonstrated that almost 20% of respondents indicated that they have no subspecialisation. Another 15% indicated that their area of subspecialisation is not recognised in their country of work. Eighty-four percent of respondents would like their area of subspecialisation in radiology to be officially recognised**.** According to the respondents, the major benefit of having their subspecialisation in radiology officially recognised is personal interest (45%).

**Conclusions:**

There is a desire for more subspecialty recognition in radiology among European radiologists. Therefore, European subspecialty diplomas in radiology fulfil a need. Furthermore, there is room for further harmonisation and implementation on a European level regarding subspecialty training and recognition in radiology.

**Critical relevance statement:**

As there is a desire for more subspecialty recognition in radiology among European radiologists, European subspecialty diplomas in radiology fulfil a need and there is still room for further harmonisation and implementation on a European level regarding subspecialty training in radiology.

**Key points:**

• Radiology has 13 subspecialties as per the European Training Curriculum for Subspecialisation.

• Currently, 15 subspecialty diplomas are offered by European subspecialty societies in radiology

• Members of the European Society of Radiology seek greater recognition of radiology subspecialties.

**Graphical Abstract:**

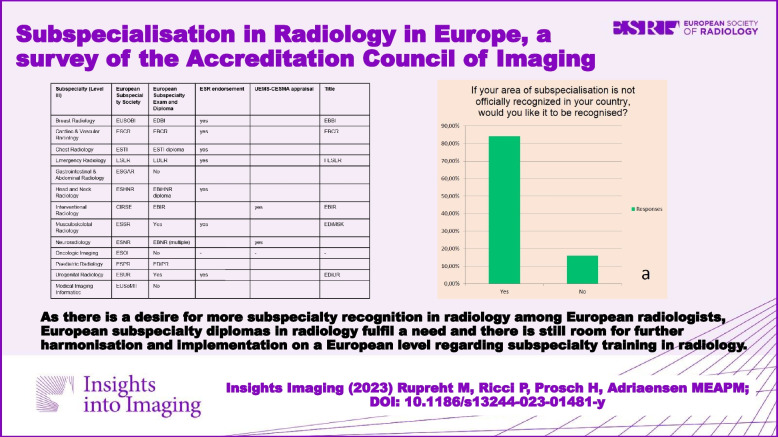

## Background

In 1994, the European Union of Medical Specialists (UEMS) adopted its “Charter on Training of Medical Specialists” with the aim to outline guiding principles for high quality medical training [1]. The first five chapters of the “Charter on Training of Medical Specialists” were common to all medical specialties, and the sixth chapter was dedicated to the specific training requirements for each individual medical discipline [1].

Currently, the European Training Curriculum for Radiology (ETCR) defines curricular contents of a five year training period and expected learning outcomes for trainees in radiology [2, 3]. The first three years of training are covering level one training and are followed by two more years of training covering a more flexible level two training with the option of more special interest rotations [2, 3]. The European Diploma in Radiology (EDiR), based on the ETCR, is provided by the European Board of Radiology (EBR) and endorsed by the European Society of Radiology (ESR) as well as UEMS. Level three training refers to a full subspecialisation training in a dedicated field of radiology [3]. The European Training Curriculum for Subspecialisation in Radiology (ETCSR) provides curricular contents for a full subspecialisation in a field of radiology [3]. As recommended by the ESR and the UEMS, at the end of level three training, objective measurement of the achieved standard should be made in line with national custom and practice and a subspecialty exam should ideally be part of the completion of training [3, 4]. ETCSR mentions thirteen fields of subspecialisation within radiology [3].

The Council of European Specialist Medical Assessment (CESMA) is an advisory body of the UEMS [4]. It was created in 2007 with an aim to provide recommendation and advice on the organisation of European examinations for medical specialists at the European level [4]. Its main roles are to promote harmonisation of European Board assessments, to provide guidelines to the Boards on the conduct of assessments, to encourage take up of Board assessments as a quality mark, and to offer an alternative to National assessments, where appropriate [4]. The CESMA stimulates the Boards and Societies that are responsible for organising the different European postgraduate medical examinations to apply for UEMS-CESMA appraisal [4]. This UEMS-CESMA appraisal is an external auditing system of the assessment that, in parallel with internal quality management systems, contributes to the continuous quality improvement of the examinations [4]. Also, the European Society of Radiology (ESR) has defined criteria for endorsement of subspecialty diplomas within radiology with the aim of homogenising the outlines of the subspecialty diplomas in radiology [5]. Examinations will be observed by the ESR, through on-site visits, at least every sixth exam or every third year [5].

The purpose of this study was to provide an overview of the existing European Subspecialty Exams and Diplomas in Radiology and their endorsement as well as to provide an insight into the status of subspecialisation in radiology in Europe.

## Methods

### Study design

The European Training Curriculum for Subspecialisation in Radiology mentions thirteen fields of subspecialisation within radiology [3]. We reviewed the member societies of the ESR Subspecialty and Allied Sciences Committee to identify the corresponding subspecialty societies. The website of each corresponding subspecialty society was checked by the first and the last author to identify information about the existence of European subspecialty exams and diplomas as well as their endorsement.

In addition, we performed a survey with regard to subspecialisation in radiology among all European radiologists who were registered as radiologist and member of the ESR (i.e. ESR full membership) in March 2021. A questionnaire was developed collaboratively by the Accreditation Council of Imaging (ACI) leadership, the last author, and the (EBR) office. The EBR is an organisation dedicated to the investigation, development and implementation of certification and accreditation activities and programmes, including examinations and other instruments of qualification certification for general and sub-specialised physicians.

The ACI is the accreditation body of the EBR and provides CME accreditation, supporting the European Accreditation Council for Continuing Medical Education (EACCME) in delivering and harmonising the highest level of CME in imaging.

The digital survey about subspecialisation in radiology was sent out on the 22nd of March 2021 to all ESR full members. The survey was open for 2 weeks. Then a reminder was sent, and members had 1 week more to respond. In line with previous studies, the online web-based software “Survey Monkey” (http://www.surveymonkey.com) was utilised to create and disseminate the survey and collect responses. In accordance with National Health Service (NHS) Health Research Authority criteria, this study did not require application for ethical approval [6].

Part one of the anonymised survey was designed to collect general information about the respondents, i.e. country of work, profession, age group, type of institution where practising the majority of the time, and radiology subspecialisation training obtained. Part two of the anonymised survey was designed to collect information about the knowledge of respondents about subspecialisation in radiology in the country of work, i.e. Which subspecialties in radiology are officially recognised in your country? How can you obtain subspecialisation in radiology in your country?; If your area of subspecialisation is not officially recognised in your country, would you like it to be recognised?; Which would be the benefits of having your subspecialty area officially recognised for you? Answers were collected as multiple-choice, ‘yes’ or ‘no’, and free text boxes were available where elaboration to answers was invited.

### Data analysis

Data was collected and tabulated independently via “Survey Monkey”. Additionally, all responses were collected in an electronic spreadsheet (Microsoft Excel, Microsoft, Redmond, VA). Results were analysed by two researchers who have been previously involved in survey studies performed by the ACI, the European Society of Musculoskeletal Radiology (ESSR), and the UEMS radiology section. Sub-analyses were performed for all countries in Europe from which we received more than 10 responses. Descriptive statistics were used to summarise multiple-choice responses, with results expressed as number of respondents and percentages. A narrative analysis was conducted on the free text answers to identify recurring themes.

## Results

According to the websites of each subspecialty society corresponding to the thirteen fields of subspecialisation within radiology mentioned in the ETCSR, 10 out of these 13 European subspecialty societies offer a European subspecialty diploma (Table 1) [7–21].

**Table 1:** Overview of existing European subspecialty exams and diplomas in radiology and their endorsement

Subspecialty (level III)	European subspecialty society	European Subspecialty Exam and Diploma	ESR endorsement	UEMS-CESMA appraisal	Title
Breast Radiology	EUSOBI	EDBI	Yes		EBBI
Cardiac & Vascular Radiology	ESCR	EBCR	Yes		EBCR
Chest Radiology	ESTI	ESTI diploma	Yes		
Emergency Radiology	ESER	EDER	Yes		FESER
Gastrointestinal & Abdominal Radiology	ESGAR	No	–	–	–
Head and Neck Radiology	ESHNR	EBiHNR diploma	Yes		
Interventional Radiology	CIRSE	EBIR		Yes	EBIR
Musculoskeletal Radiology	ESSR	Yes	Yes		EDiMSK
Neuroradiology	ESNR	EBNR (multiple)		Yes	
Oncologic Imaging	ESOI	No	–	–	–
Paediatric Radiology	ESPR	EDiPR			
Urogenital Radiology	ESUR	Yes	Yes		EDiUR
Medical Imaging Informatics	EUSoMII	No	–	–	–

List of subspecialties in radiology (according to the European Training Curriculum for Subspecialisation in Radiology), their corresponding European subspecialty society, the name of an existing European Subspecialty Exam and Diploma, endorsement by the European Society of Radiology and/or the Council of European Specialist Medical Assessment, and title to be added behind the name of successful candidates indicating full subspecialisation in radiology as allowed by the subspecialty society

The European Board of Neuroradiology offers multiple diplomas. As mentioned on its website, the European Board of Neuroradiology—Diagnostic and Interventional (EBNR) is a professional organisation dedicated to organising exams and issuing certificates for the European Diploma in Neuroradiology and higher qualifications in all diagnostic and interventional neuroradiology subspecialties [21]. Currently, the EBNR is providing six European Diplomas in the field of neuroradiology, the European Diploma in Neuroradiology (EDiNR), the European Diploma in Pediatric Neuroradiology (EDiPNR), the European Diploma in Interventional Neuroradiology (EDiINR), the European Diploma in Spine Radiology—diagnostic (EDiSR—diagnostic), the European Diploma in Spine Radiology—interventional (EDiSR—interventional), and the European Diploma in Head and Neck Neuroradiology (EDiHNNR). Therefore, there are two diplomas for head and neck radiology. One offered by the ESHNR, and one offered by the ESNR.

Currently, at least 7 out of the 10 European subspecialties societies in radiology offering a European subspecialty diploma obtained ESR endorsement (Table 1). Two out of 10 obtained UEMS-CESMA endorsement (Table 1). Six out of the 10 European subspecialty societies in radiology allow successful candidates to add a title behind their name indicating their full subspecialisation in radiology (Table 1).

The digital survey about subspecialisation in radiology was sent out on the 22nd of March 2021 to all European radiologists who were registered as radiologist and member of the ESR (i.e. ESR full membership) in March 2021 (*n* = 13,531). 823 answers were received, giving a response rate of 6%.

**Fig. 1:**
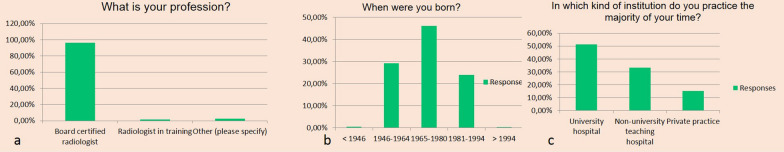
Distribution of completed surveys stratified by profession (**a**), by age (**b**) and by main type of practice (**c**) of respondents

**Fig. 2:**
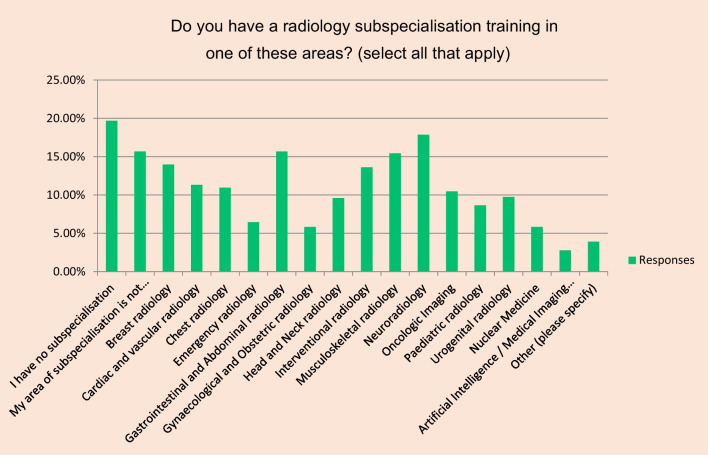
Distribution of completed surveys by self-reported area(s) of subspecialisation in radiology My area of subspecialisation is not ...equals My area of subspecialisation is not recognised in my country Artificial Intelligence / Medical Imaging... equals Artificial Intelligence / Medical Imaging informatics

Answers were received from 43 different countries. Number of respondents per country varied from 1 to 145. Most answers were received from Italy (*n* = 145), UK (*n* = 131), Germany (*n* = 111) and Spain (*n* = 98) (Table 2). Most respondents were board certified radiologists (96%, *n* = 791) and were between 40 and 55 years old (46%, *n* = 380). About half of the respondents work most of their time in a university hospital (*n* = 423) (Fig. 1). Almost 20% of respondents indicated that they have no subspecialisation (*n* = 162). Another 16% indicated that their area of subspecialisation is not recognised in their country of work (*n* = 129) (Fig. 2).

**Table 2:** Distribution of completed surveys stratified by country of work

Country of work	Number of responses	Percentages of all respondents
Albania	2	0.2
Austria	20	2.4
Armenia	2	0.2
Belarus	7	0.9
Belgium	50	6.1
Bosnia and Herzegovina	1	0.1
Bulgaria	2	0.2
Croatia	8	1.0
Cyprus	1	0.1
Czech Republic	2	0.2
Denmark	5	0.6
Estonia	1	0.1
Finland	2	0.2
France	11	1.3
Georgia	5	0.6
Germany	111	13.5
Greece	17	2.1
Hungary	14	1.7
Iceland	3	0.4
Ireland	6	0.7
Italy	145	17.6
Kosovo	1	0.1
Latvia	12	1.5
Lithuania	7	0.9
Luxembourg	1	0.1
Malta	1	0.1
Republic of Montenegro	1	0.1
The Netherlands	13	1.6
Norway	2	0.2
Poland	12	1.5
Portugal	9	1.1
Romania	19	2.3
Russia	4	0.5
Serbia	9	1.1
Slovakia	4	0.5
Slovenia	12	1.5
Spain	98	11.9
Sweden	41	5.0
Switzerland	13	1.6
Turkey	8	1.0
Ukraine	9	1.1
UK	131	15.9
Uzbekistan	1	0.1

**Fig. 3:**
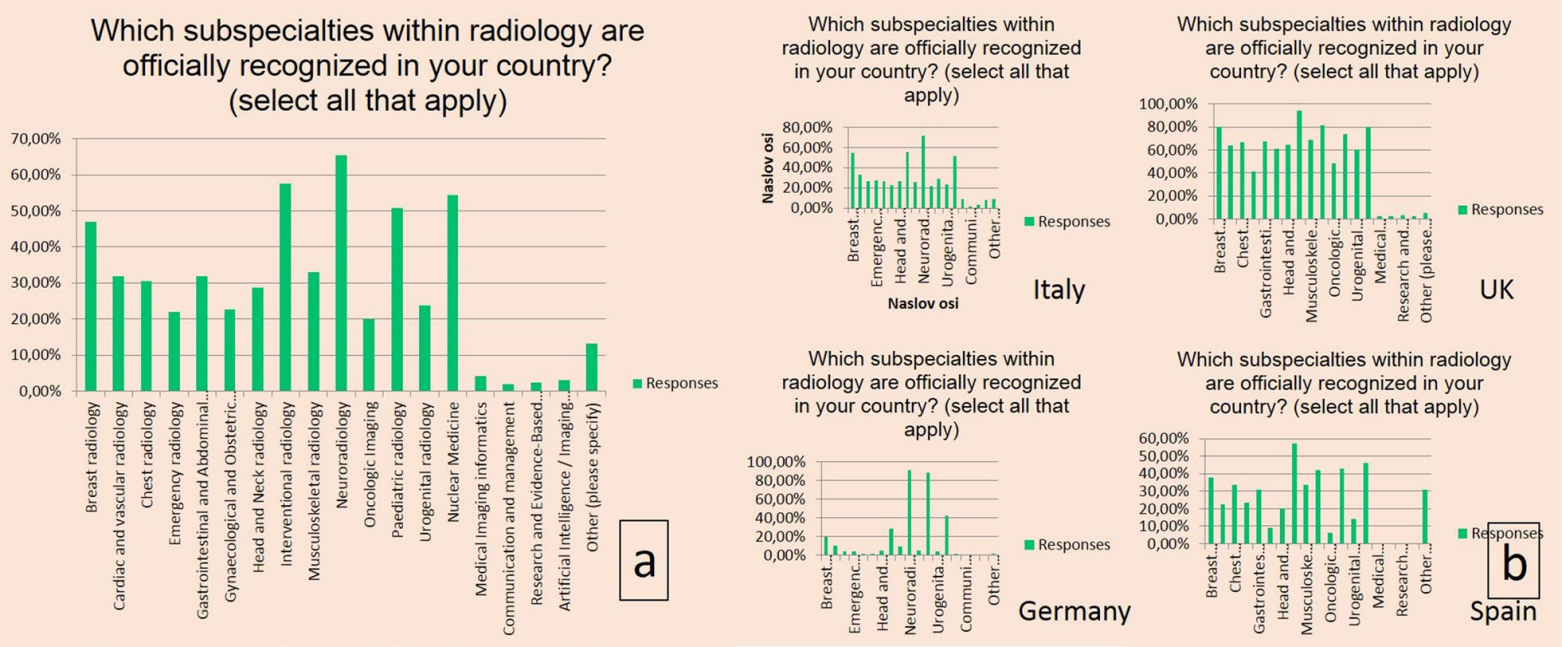
Overview of self-reported officially recognised subspecialties in radiology in the country of work of the respondent. All countries (**a**). Sub-analyses per four countries with largest numbers of respondents (**b**)

Overall results showed that the top five of officially recognised subspecialties, according to the respondents, are neuroradiology, interventional radiology, nuclear medicine, paediatric radiology, and breast radiology (Fig. 3a). Sub-analyses per four countries with largest numbers of respondents showed a heterogeneity in answers regarding the official recognition of subspecialties in radiology within a country (Fig. 3b).

**Fig. 4:**
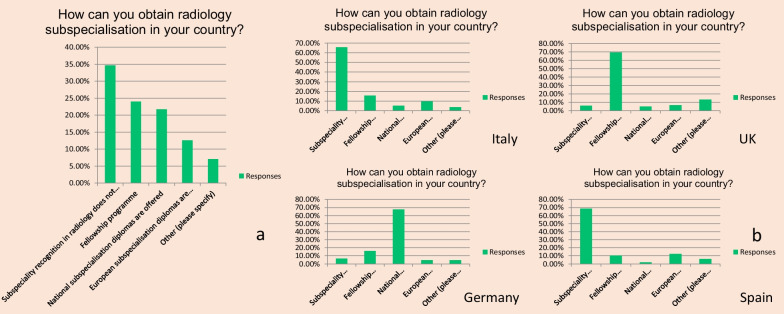
Overview of self-reported ways to obtain an officially recognised subspecialty in radiology. All countries (**a**). Sub-analyses per four countries with largest numbers of respondents (**b**) Subspeciality recognition in radiology does not... equals Subspeciality recognition in radiology does not exist in my country European subspecialisation diplomas are... equals European subspecialisation diplomas are recognised in my country

Overall results showed that the three most common ways to obtain subspecialisation in radiology are fellowship programmes, national subspecialisation diplomas and European subspecialisation diplomas (Fig. 4a). Sub-analyses per country showed that apparently in Italy and Spain subspeciality recognition in radiology does not exist. In the UK, there are national fellowship programmes to obtain subspecialisation. And in Germany, there are national subspecialisation diplomas (Fig. 4b).

**Fig. 5:**
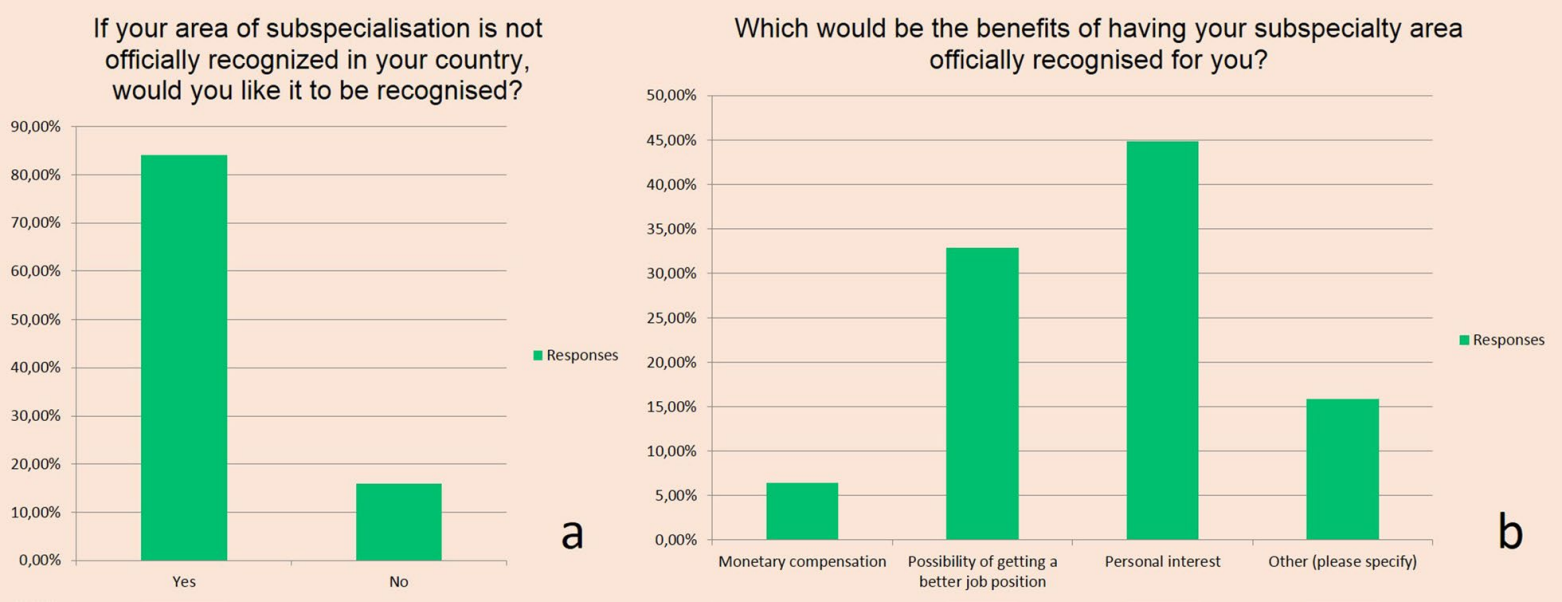
Desire to have subspecialty in radiology officially recognised (**a**). Answers of respondents regarding the potential benefit of official recognition of their subspecialty (**b**)

Most respondents (84%, *n* = 617) would like their area of subspecialisation in radiology to be officially recognised **(**Fig. 5a). According to the respondents, the major benefit of having their subspecialisation in radiology officially recognised is personal interest (45%, *n* = 308) (Fig. 5b). Several other benefits of having their subspecialisation in radiology officially recognised were highlighted by the respondents as well. A lot of respondents mentioned the benefit of quality improvement and better patient care. Other benefits mentioned were better training and improved workflow. “Across the board more subspecialised work in radiology would lead to higher medical quality and faster work.” However, another respondent mentioned “less workload” as a benefit of subspecialisation in radiology. Public relations and marketing was mentioned as well. “I would be able to demonstrate my expertise to patients and referring physicians.” Furthermore, recognition was indicated as a benefit of having their subspecialisation in radiology officially recognised. “Official recognition can serve as a quality mark and result in equality of recognition of all different subspecialities in radiology.” Easier recruiting of colleagues within a certain subspecialty was also mentioned as a benefit of official subspecialisation, as was the benefit of better lobbying opportunities, like better representation in national discussions on imaging. Also, dedicated organisation and negotiation with government/health care organisations/insurance companies for specific items in subspeciality were mentioned. Finally, protection against turf battles was seen as a benefit of officially recognised subspecialisation within radiology, such as adding difficulty for other medical specialties to "steal" Interventional radiology procedures from interventional radiologists or to claim competence of cardiac CT/MRI for Radiology.

## Discussion

In 1984, the first European Specialist Medical Diploma Examination was established [4]. This was the European Diploma of Anaesthesiology [4]. Today, more than 30 disciplines have European Examinations [4]. Within the field of Radiology, we currently have the EDiR and 15 subspecialty diplomas provided by European subspecialty societies in radiology [8–23]. Among these, 6 are provided by one subspecialty society (EBNR) [21].

One of the limitations of the survey was the relatively low response rate of only 6%. However, regardless of the low response rate, we received some interesting results. We expected that the answers per country would indicate whether a subspecialty within radiology is recognised or is not recognised in a certain country. Combined responses per subspecialty per country were therefore expected to be either at 100% or stay close to zero. However, we observed a heterogeneity in the responses within countries. One of the possible explanation for the heterogeneity could be a possible lack of familiarity amongst respondents with the recognition of subspecialties in radiology in their respective countries. This possible lack of familiarity could encourage medical societies in each country to pro-actively disseminate this information to radiologists. Alternatively, a follow-up survey could be performed targeting national experts with regard to formal national training, recognition and continuing medical education and continuing professional development of subspecialties in radiology. The above-mentioned heterogeneity probably could also reflect different types of European (ESR endorsement versus UEMS-CESMA endorsement) and national endorsements of subspecialty recognition within radiology. However, this heterogeneity should motivate further harmonisation and collaboration amongst societies, particularly umbrella organisations, with the goal of implementing and recognising radiology subspecialty training in Europe, which is also in accordance with the desire of the majority of respondents to have their area of subspecialisation in radiology officially recognised.

For two of the subspecialties without formal subspecialty exams and diplomas, i.e. European Society of Gastrointestinal and Abdominal Radiology (ESGAR) and European Society of Oncologic Imaging (ESOI), a large percentage (26%, *n* = 215) of respondents claim to have one or the other subspecialty. Both ESGAR and ESOI offer a three-month exchange programme for fellowships or subspecialisation training, supported by ESR and European School of Radiology [12, 17]. Both together with the European Society of Medical Imaging Informatics (EUSoMII) represent fast growing fields regarding patient’s needs and technological development, indicating the potential for official subspecialty diplomas.

ESR recognises many strong arguments and listed several reasons in favour of subspecialisation, such as information overload, rapid development, clinicians in secondary and tertiary centres, are all specialised, technological developments, the need for the most accurate diagnosis, and increased appreciation for translational research and evidence value-based health care [24, 25]. Also, the education in different fields of radiology (for both general radiologists and future subspecialists) will benefit from the presence of officially subspecialised radiologists [24, 25]. One should keep in mind, however, that in the future, both general radiological knowledge and subspecialised radiological knowledge will still be needed to maintain a high standard of radiological expertise during all hours of 24/7 service.

## Conclusions

To conclude, there is a desire for more subspecialty recognition in radiology among European radiologists. Therefore, European subspecialty diplomas in radiology fulfil a need. Furthermore, there is room for further harmonisation and implementation on a European level regarding subspecialty training and recognition in radiology.

## Data Availability

The dataset used and analysed is available from the corresponding author on reasonable request.

## Abbreviations

ACI: Accreditation Council of Imaging
CESMA: Council of European Specialist Medical Assessment
CIRSE: Cardiovascular and Interventional Radiological Society of Europe
EACCME: European Accreditation Council for Continuing Medical Education
EBBI: European Board of Breast Imaging
EBCR: European Board of Cardiovascular Radiology
EBiHNR: European Board in Head and Neck Radiology
EBIR: European Board of Interventional Radiology
EBR: European Board of Radiology
EBNR: European Board of Neuroradiology – Diagnostic and Interventional
EDBI: European Diploma in Breast Imaging
EDER: European Diploma in Emergency Radiology
EDiHNNR: European Diploma in Head and Neck Neuroradiology
EDiINR: European Diploma in Interventional Neuroradiology
EDiMSK: European Diploma in MusculoSKeletal Radiology
EDiNR: European Diploma in Neuroradiology
EDiPNR: European Diploma in Pediatric Neuroradiology
EDiPR: European Diploma in Paediatric Radiology
EDiR: European Diploma in Radiology
EDiSR – diagnostic: European Diploma in Spine Radiology – diagnostic
EDiSR – interventional: European Diploma in Spine Radiology – interventional
EDiUR: European Diploma in Urogenital Radiology
ESCR: European Society of Cardiovascular Radiology
ESER: European Society of Emergency Radiology
ESGAR: European Society of Gastrointestinal and Abdominal Radiology
ESHNR: European Society of Head and Neck Radiology
ESNR: European Society of Neuroradiology
ESOI: European Society of Oncologic Imaging
ESPR: European Society of Paediatric Radiology
ESR: European Society of Radiology
ESSR: European Society of Musculoskeletal Radiology
ESTI: European Society of Thoracic Imaging
ESUR: European Society of Urogenital Radiology
ETCR: European Training Curriculum for Radiology
ETCSR: European Training Curriculum for Subspecialisation in Radiology
EUSOBI: European Society of Breast Imaging
EUSoMII: European Society of Medical Imaging Informatics
FESER: European Fellow of Emergency Radiology
NHS: National Health Service
UEMS: European Union of Medical Specialists
