# Correction: Subspecialisation in radiology in Europe, a survey of the accreditation council of imaging

**DOI:** 10.1186/s13244-023-01565-9

**Published:** 2023-11-17

**Authors:** Mitja Rupreht, Paolo Ricci, Helmut Prosch, Miraude E. A. P. M. Adriaensen

**Affiliations:** 1 Radiology Department, UMC Maribor, Ljubljanska 5, 2000 Maribor, Slovenia; 2 Medical Faculty, University of Maribor, Maribor, Slovenia; 3 Department of Radiology and Nuclear Medicine, Maastricht UMC+, Maastricht, The Netherlands; 4 Department of Radiological, Oncological and Pathological Sciences, Sapienza University of Rome, Rome, Italy; 5 Department of Biomedical Imaging and Image-Guided Therapy, Medical University of Vienna, Vienna, Austria; 6 Department of Medical Imaging, Zuyderland Medical Center, Sittard-Geleen, Heerlen, Brunssum, Kerkrade The Netherlands

**Correction: Insights Imaging 14, 159 (2023)**

**https://doi.org/10.1186/s13244-023-01481-y**

In the original article [1], the European Society of Radiology (ESR) endorsement of the European Board of Interventional Radiology (EBIR) subspecialty diploma was erroneously not disclosed.

This necessitates the following corrections:

1) In both the Results section and the Results sub-section of the Abstract, the phrase “[…] 7 out of the 10 European subspecialties […]” should state 8 instead of 7.

2) In Table 1 and, consequently, in the graphical abstract that uses Table 1, this endorsement has now been noted.
